# Seroprevalence of Zika in Brazil stratified by age and geographic distribution

**DOI:** 10.1017/S0950268823001814

**Published:** 2023-11-15

**Authors:** Viviane Fongaro Botosso, Alexander Roberto Precioso, Annelies Wilder-Smith, Danielle Bruna Leal de Oliveira, Fabyano Bruno Leal de Oliveira, Cairo Monteiro De Oliveira, Camila Pereira Soares, Lucyana Trindade Leal Oliveira, Ralyria Mello Vieira dos Santo, Carla Lilian de Agostini Utescher, Francisco Antonio Bezerra Coutinho, Eduardo Massad

**Affiliations:** 1 Instituto Butantan, São Paulo, Brazil; 2 Heidelberg Institute of Global Health, University of Heidelberg, Heidelberg, Germany; 3 School of Medicine, University of São Paulo, São Paulo, Brazil; 4 Fundação Getúlio Vargas, Rio de Janeiro, Brazil

**Keywords:** arboviruses, emerging infections, epidemiology, mathematical modelling, Zika virus

## Abstract

Congenital Zika is a devastating consequence of maternal Zika virus infections. Estimates of age-dependent seroprevalence profiles are central to our understanding of the force of Zika virus infections. We set out to calculate the age-dependent seroprevalence of Zika virus infections in Brazil. We analyzed serum samples stratified by age and geographic location, collected from 2016 to 2019, from about 16,000 volunteers enrolled in a Phase 3 dengue vaccine trial led by the Institute Butantan in Brazil. Our results show that Zika seroprevalence has a remarkable age-dependent and geographical distribution, with an average age of the first infection varying from region to region, ranging from 4.97 (3.03–5.41) to 7.24 (6.98–7.90) years. The calculated basic reproduction number, 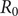 , varied from region to region, ranging from 1.18 (1.04–1.41) to 2.33 (1.54–3.85). Such data are paramount to determine the optimal age to vaccinate against Zika, if and when such a vaccine becomes available.

## Introduction

The emergence of Zika virus in the Americas led to the declaration of a public health emergency of international concern in January 2016 due to its causal association with congenital Zika syndrome (CZS) as a result of maternal infection [1, 2]. Although Zika virus infections (ZVI) had already been described in the 1940s in Africa, the risk of birth defects only came to light during the explosive outbreak in Brazil in 2016 [3]. It appears that virus attenuation led to the emergence of CZS, whereby the virus has evolved to be less pathogenic not resulting in foetal death but in foetal anomalies [4–7]. The emergence of severe birth defects prompted an accelerated search for Zika vaccines [8, 9]. Due to the decline in cases towards 2017, Phase 3 trials are no longer feasible [8]. The rapid geographic spread of ZVI in Brazil and beyond led to high seroprevalence rates with high population-level immunity, thus effectively ending the public health emergency of international concern [10].

The spread of ZVI was not homogeneous and was mainly driven by mobility patterns and population densities [10]. Re-emergence is highly likely, as ZVI have not disappeared with occasional outbreaks and sporadic cases still being reported [11–14]. The explosive outbreak that resulted in more than 280,000 reported cases in 2016 has stabilized at endemic levels of around 20,000 new cases per year in the subsequent years (http://tabnet.datasus.gov.br/cgi/tabcgi.exe?sinannet/cnv/zikabr.def).

If and when a Zika vaccine becomes available, vaccine introduction needs to take into account age-stratified seroprevalence rates and geographic distribution of seroprevalence. The objective of our study is to determine Zika seroprevalence rates stratified by age and geolocation in Brazil.

## Methods

### Study design

The Phase 3 trial to evaluate the efficacy and safety of a live attenuated tetravalent dengue vaccine (DEN-03-IB) developed by the National Institute of Health, United States, was carried out by the Butantan Institute in Brazil from 2016 to 2019 (ClinicalTrials.gov Identifier: NCT02406729) [15]. All study subjects had blood samples taken at baseline. As the DEN-03-IB study was designed before the Zika outbreak in Brazil, an amendment to the ethics approval was sought in April 2016 to include the study of other arboviruses, including the Zika virus. The National Ethics Council (CONEP) approved this amendment on 1 July 2016 (CAAE: 44462915.8.1001.0068).

All blood samples were collected before vaccination over the recruitment period from 2016 to 2019, with an age de-escalating approach starting with adults (18–59 years old), adolescents (7–17 years old) followed by children (2–6 years old). Samples were representative of the period from 2016 to 1,019 and of different geolocations representing geographic regions of Brazil.

### Sampling methods

The number of individuals sampled within each age class, 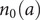 , was estimated using standard theory [16].
(1)

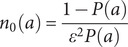

where 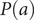 is the a priori estimate of seroprevalence and 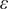 is set for the desired level of precision (assumed as 0.2). In the absence of suitable age-stratified Zika serology in Brazil, the expected proportion of seropositive individuals by age was estimated from the notified number of cases, reported to the Ministry of Health (http://tabnet.datasus.gov.br/cgi/tabcgi.exe?sinannet/cnv/zikabr.def). Since the communities studied were relatively small, a finite population correction factor was applied to sample sizes [16] as follows:
(2)

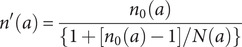

The sampling could be described succinctly as a 2-level cluster sample: individuals were sampled within families within randomly selected administrative regions [16]. The number of necessary dwellings to be visited within the community is such that by chance the desired sample size for each age class would be achieved, 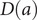 . It was calculated by:
(3)

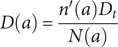

where 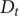 is the total number of dwellings in the town, 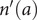 is the number of individuals within the age class corrected to a finite population, and 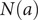 is the number of individuals of age 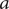 known from census records [17].

### Blood sample collection

**Figure 1.**
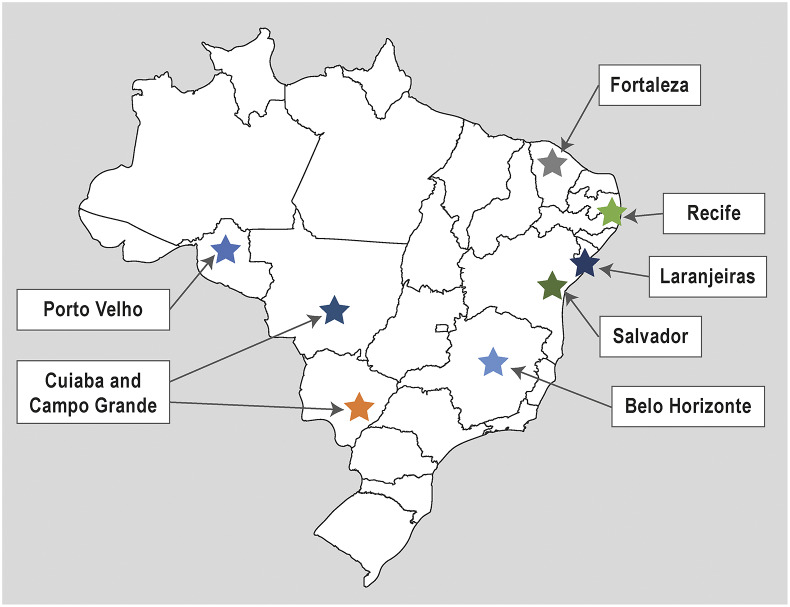
Sites of sample collection.

Blood samples from 5,300 subjects, aged 2 to 59 years old, were collected from July 2016 to June 2019 from seven sites in Brazil. Of these seven sites, four were from the North-eastern region of the country (Recife, Salvador, Fortaleza, and Laranjeiras), one from the South-eastern region (Belo Horizonte), one from the Northern region (Porto Velho), and two from the Central region (Cuiabá and Campo Grande) (Figure 1). Samples were collected one day before the dengue vaccination. Pregnant and lactating women, immunocompromised persons, and persons with underlying comorbidities were excluded.

Serum samples were taken and kept on ice during transport and then stored at − 80 °C before serological testing.

## Diagnostic assay

We tested Zika IgG-specific antibodies in serum, using a commercial indirect anti-Zika IgG ELISA (Zika-V IgG ELISA–AdvaGen), following exactly the manufacturers’ instructions. Briefly, in this technique, the Zika recombinant ∆NS-1 antigen is adhered to a solid surface. In order to remove the cross-reactivity with the dengue virus, the patient’s serum was previously adsorbed with dengue 1, 2, 3, and 4 ∆NS-1 antigens before being added to the reaction. The assay was conducted as per protocol. The serum was considered positive if the optical density (O.D.) at 450 nm is ≥0.272, indeterminate if 0.217 > O.D. 450 nm < 0.272, and negative if the O.D. is ≤0.217. This kit was chosen because of its high specificity for the detection of anti-Zika antibodies, even in dengue endemic areas [18–25]. Table 1 shows sensitivity, specificity, positive predictive value, and negative predictive value for the ELISA test used.

**Table 1.** Sensitivity, specificity, positive predictive value, and negative predictive value parameters for performance assessment of ELISA ZIKA-v IgG

	95%CI
Sensitivity (%)	90.7 (80.98–98.57)
Specificity (%)	94.12 (86.8–98.06)
PPV (Positive predictive value) (%)	90.7 (88.3–93.1)
NPV (Negative predictive value) (%)	94.12 (92.3–95.8)

*Note*: Cutoff 0,2,295.

To determine cross-reaction with other flaviviruses, 35 dengue positive samples (confirmed by PRNT90) from São José do Rio Preto city, state of São Paulo, and 40 samples from volunteers who had received a yellow fever vaccine from Salvador city, Bahia state, were used. No cross-reactivity was observed.

## Calculation of age-dependent seroprevalence and the average age of the first infection

We adjusted a continuous curve, given by Equation (4), to the proportion of seropositive individuals to Zika, 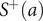 :
(4)

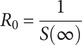

Parameters *k*_1_ to *k*_3_ have no biological meaning and are just fitting parameters. The model was fitted to data by the least-square method.

From the seroprevalence curve, it follows that the age-dependent force of infection (incidence density), 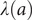 , is given by [26]:
(5)

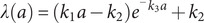

where the parameters *k_i_* are the same as in Equation (4), fitted to the data for each site.

Given the seroprevalence curve (Equation (4)) and the force of infection (Equation (5)), it follows that the average age of the first infection, 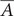 , is given by:
(6)

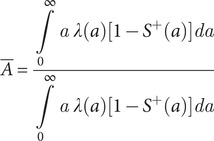


**Figure 2.**
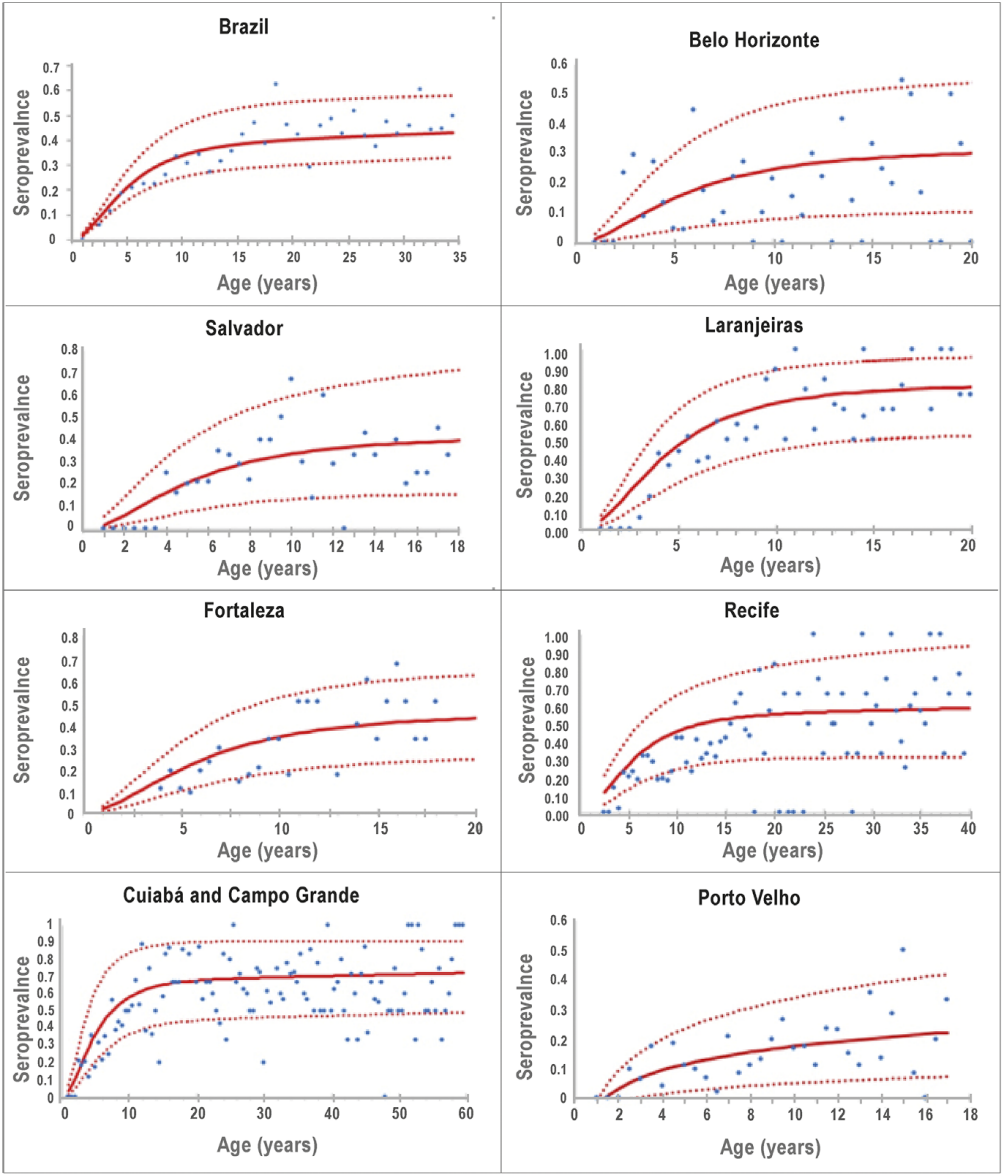
Seroprevalence data (dots) and the continuous function (Equation (4)) adjusted to the data of Brazil as a whole and to the seven collection sites. The continuous line represents the average and the dotted lines represent the 95% confidence interval.

Figure 2 shows the adjusted seroprevalence profile (Equation (4)) of the country as a whole and to the seven collection sites.

Note that it appears from Figure 2 that the seroprevalence in children younger than 2 years age is practically zero. This in fact is a fitting artefact because the cohorts were sampled from age 2 upwards. In some places, the sample size was too small to allow a more accurate fitting.

## Results

From the total of 5,300 serum samples, 1,531 were positive (28.9%) for Zika, 3,491 (65.9%) were negative, and 278 (5.2%) were undetermined (grey zone). The distribution of Zika virus IgG seroprevalence per geographic location, as well as the average age of the first infection, is shown in Table 2.

**Table 2.** Sample size, seroprevalence, and average age of the first infection for each site

Site	Sample size	Seroprevalence^a^	Average age of infection^b^	R_0_^c^
Brazil	5,108^d^	0.35 (0.26–0.48)	7.24 (6.98–7.90)	1.54 (1.35–1.92)
Belo Horizonte	817	0.22 (0.07–0.41)	6.33 (5.43–6.33)	1.28 (1.08–1.69)
Salvador	494	0.29 (0.11–0.52)	5.66 (5.12–6.03)	1.41 (1.12–2.08)
Laranjeiras	574	0.57 (0.35–0.74)	7.27 (3.32–5.36)	2.33 (1.54–3.85)
Fortaleza	345	0.30 (0.17–0.46)	6.32 (5.48–7.09)	1.43 (1.21–1.85)
Recife	1,110	0.47 (0.25–0.69)	6.72 (6.40–7.28)	1.89 (1.33–3.23)
Cuiaba and Campo Grande	1,017	0.40 (0.30–0.72)	4.97 (3.03–5.41)	1.67 (1.43–3.57)
Porto Velho	751	0.15 (0.04–0.29)	5.90 (5.54–6.02)	1.18 (1.04–1.41)

a according to Equation (3).

b according to Equation (5).

c according to Equation (7).

d excluding undetermined.

The age-dependent seroprevalence for Brazil stabilized at the average value of 35% (95% CI 26–48%), which means that the seropositive proportion for the country as a whole did not change above the age of 35 years. The relationship between the fraction of people remaining susceptible at the end of the epidemic, 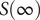 , (that is, calculated for very large ages) and the basic reproduction number, 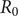 is given by [27]:
(7)

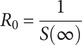

Its value for Brazil as a whole is 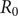 =1.54 (1.35–1.92), which agrees with the estimation of Villela et al. [28] of 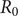 for Zika virus, who found the value of *R*_0_ = 1·25 (1·18–1·36) for Brazil. The values of the basic reproduction number for the other sites are given in Table 2.

**Figure 3.**
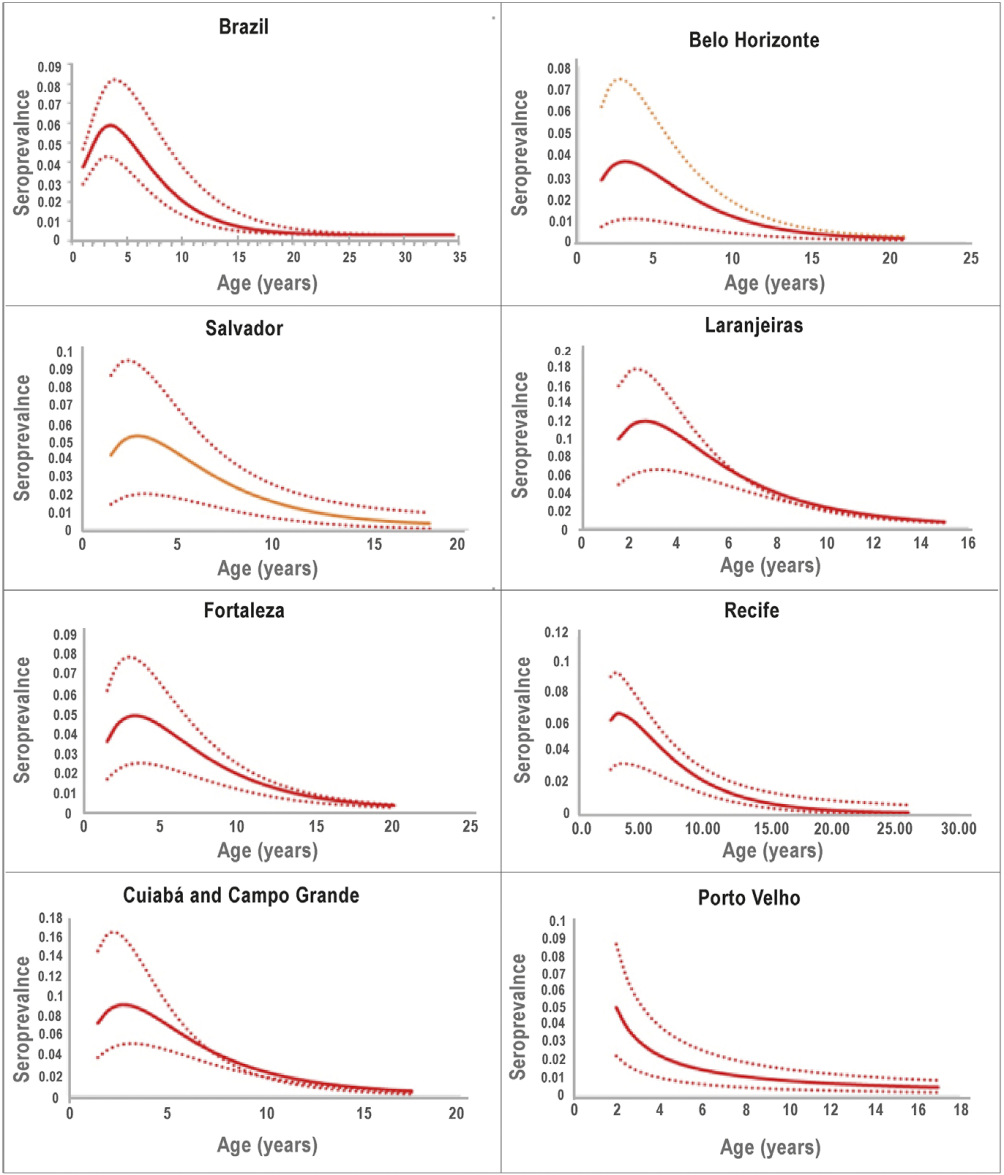
Age-dependent forces of infection as calculated according to Equation (6). The continuous lines represent the average and the dotted lines represent the 95% confidence intervals.

Figure 3 shows the age-dependent forces of infection for Brazil as a whole and for the other 7 collection sites.

Note that for the country as a whole the force of infection declines to zero after the age of 35 years because, as mentioned above, the proportion of seropositive persons above this age did not change. In addition, it can be noted that the force of infection for children below age 2 years is not shown in the figure. This is due to the fact that children below age 2 were not sampled in this study.

Table 3 shows the correlation between the human development index (HDI), a proxy for the socioeconomic level of each site, and the basic reproduction number, 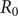 , which is a proxy for the intensity of transmission of Zika virus in each site.

**Table 3.** Human development index and basic reproduction number

SITE	HDI	R_0_
Belo Horizonte	0.81	1.28
Salvador	0.70	1.41
Laranjeiras	0.64	2.33
Fortaleza	0.75	1.43
Recife	0,78	1.89
Cuiaba and Campo Grande	0.75	1.67
Porto Velho	0.73	1.18

*Note*: Pearson correlation coefficient *r* = 0.49. Note that the intensity of transmission correlates negatively with the socioeconomic level of each studied site (*r* = 0.49).

## Discussion

The objective of our study is to determine the age-dependent seroprevalence of Zika infection in Brazil. Our results show that there is a remarkable age-dependent distribution of seroprevalence, with an average age of the first infection varying from region to region, ranging from 4 (3.03–5.41) to 7 (6.98–7.90) years.

The calculated basic reproduction number, 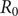 , varied from region to region, ranging from 1.18 (1.04–1.41) to 2.33 (1.54–3.85). These results are consistent with previous estimations [28].

The calculation of the age-dependent seroprevalence profile is central to the estimation of the optimal age to vaccinate against Zika, if and when a Zika vaccine becomes available. As shown by Massad et al. [26] for the case of rubella and by Maier et al. [29] for the case of dengue, age-dependent seroprevalence rates have guided decisions for rubella and dengue vaccine introductions. The optimal age to vaccinate should be targeted to the age range below the average age of the first infection. Our results therefore provide age estimates that can guide the optimal age for introducing a Zika vaccine.

By using a simple Ross–Macdonald model, it is possible to project the expected number of cases for the post-epidemic period. Preliminary results suggest that Zika could well return around 2026 with a new epidemic wave [30]. This conjecture, however, needs a more elaborate analysis.

One important limitation of this study is the fact that after the 2016-2017 outbreak, Zika did not completely stabilize and remained since only approximately in a steady state. Since a steady state is a condition for the proper calculation of the average age of the first infection, our results, with respect to this, are only an approximation.

Our findings show that the geographic distribution of seroprevalence mirrors the incidence of reported cases in Brazil from 2016 to 2017 with the highest number of reported cases in the North-eastern and Central regions. These two regions accounted for around 70% of the total cases reported in the country. ((http://tabnet.datasus.gov.br/cgi/tabcgi.exe?sinannet/cnv/zikabr.def). The North-eastern region, especially the semiarid region in which the average range of rainfall is from 500 to 800 mm, is considered less developed. In addition, a significant number of municipalities are characterized by low human development indexes (HDIs). Consequently, these populations are socioeconomically vulnerable with a monthly family income around 5 to 10 times lower than in regions with higher HDIs. Previous studies showed higher Zika virus infection rates in lower social strata from North-eastern cities of Brazil, which may reflect the precarious living conditions, with the lack of basic sanitation and water storage resulting in greater vector abundance [31, 32]. Our results, which show a negative correlation between the HDI and the intensity of Zika virus transmission in the studied regions, confirm these previous findings.

Finally, it should be mentioned that the age-stratified seroprevalence of Zika is similar to the age-stratified seroprevalence of dengue. Although the Zika virus epidemic at the time of the study was explosive across all ages and then rapidly declined, Zika remains endemic in several regions of the country up to this day (http://www.portalsinan.saude.gov.br/).

In summary, our results can help identify the target population for a future vaccination programme.

## Data Availability

All data, code, and other materials in this paper are available to readers without undue barriers to access, as per request.
